# Insulin-Like Growth Factor Receptor Signaling is Necessary for Epidermal Growth Factor Mediated Proliferation of SVZ Neural Precursors *in vitro* Following Neonatal Hypoxia–Ischemia

**DOI:** 10.3389/fneur.2014.00079

**Published:** 2014-05-26

**Authors:** Dhivyaa Alagappan, Amber N. Ziegler, Shravanthi Chidambaram, Jungsoo Min, Teresa L. Wood, Steven W. Levison

**Affiliations:** ^1^Department of Neurology and Neuroscience, New Jersey Medical School, Rutgers University, Newark, NJ, USA

**Keywords:** cell proliferation, stem cell niche, growth factors, central nervous system, regeneration

## Abstract

In this study, we assessed the importance of insulin-like growth factor (IGF) and epidermal growth factor (EGF) receptor co-signaling for rat neural precursor (NP) cell proliferation and self-renewal in the context of a developmental brain injury that is associated with cerebral palsy. Consistent with previous studies, we found that there is an increase in the *in vitro* growth of subventricular zone NPs isolated acutely after cerebral hypoxia–ischemia; however, when cultured in medium that is insufficient to stimulate the IGF type 1 receptor, neurosphere formation and the proliferative capacity of those NPs was severely curtailed. This reduced growth capacity could not be attributed simply to failure to survive. The growth and self-renewal of the NPs could be restored by addition of both IGF-I and IGF-II. Since the size of the neurosphere is predominantly due to cell proliferation we hypothesized that the IGFs were regulating progression through the cell cycle. Analyses of cell cycle progression revealed that IGF-1R activation together with EGFR co-signaling decreased the percentage of cells in G1 and enhanced cell progression into S and G2. This was accompanied by increases in expression of cyclin D1, phosphorylated histone 3, and phosphorylated Rb. Based on these data, we conclude that coordinate signaling between the EGF receptor and the IGF type 1 receptor is necessary for the normal proliferation of NPs as well as for their reactive expansion after injury. These data indicate that manipulations that maintain or amplify IGF signaling in the brain during recovery from developmental brain injuries will enhance the production of new brain cells to improve neurological function in children who are at risk for developing cerebral palsy.

## Introduction

Many studies over the past decade have touted the regenerative potential of the endogenous neural precursors (NPs) of the subventricular zone (SVZ) following injury. Although encouraging, the extent of cell replacement, both in terms of the numbers of cells produced and the variety of cell types generated is limited. However, by understanding the mechanisms that regulate the growth of SVZ cells, therapeutic strategies can be designed to achieve a more significant level of regeneration after CNS injury. In an earlier study, we used flow cytometry on SVZ cells isolated acutely after neonatal hypoxia–ischemia (H–I) and showed that epidermal growth factor receptor (EGFR) expression increased significantly on primitive NPs of the SVZ during recovery from neonatal H–I. Furthermore, we showed that pharmacologically inhibiting the EGFR reduced the expansion of the NPs that normally occurs (1).

Despite wide acceptance that the EGF receptor is a key mitogen for NPs, there is increasing evidence that other ligands are central regulators of NP cell cycle progression. Two key regulators are the insulin-like growth factors (IGF-I and IGF-II). The critical importance of IGFs for neural development was shown in studies that produced genetically engineered mice with disrupted IGF-I gene expression. These gene targeted mice showed profound *in utero* and postnatal growth retardation (2). IGF-II knockouts showed similar growth retardation with onset earlier during gestation and, like the IGF-1 deficient mice, showed a 40% reduction in body mass at birth (3). Homozygous IGF-1R null mice have more profound developmental deficits at the end of gestation, and they do not survive beyond a few hours after birth (2).

Complementary studies using transgenic mice where IGF-I was expressed downstream of the regulatory elements for nestin that are specifically active in NPs (4), have provided additional insights into the roles of IGF-I in CNS neural development. In these studies, overexpressing IGF-I increased the fraction of embryonic day 14 neuroepithelial cells in S phase by over 15% compared to wild type animals. When these transgenic mice were analyzed postnatally, there was a significant increase in the total number of neurons generated accompanied by a 26% reduction in the number of apoptotic cells compared to age matched wild type animals. These results suggested that, *in vivo*, IGF-I promotes the proliferation of NPs during the genesis of the CNS and that IGF-I also promotes survival (5).

*In vitro* studies on the effects of IGF-I on NPs have been somewhat contradictory. Arsenijevic et al. (6) showed that embryonic day 14 murine striatal NPs failed to proliferate in serum free, insulin free media even when supplemented with EGF and FGF-2. However, upon IGF-I addition, they grew effectively and formed neurospheres in culture. IGF-I stimulation as short as 24 h, in the constant presence of EGF, was sufficient to produce neurospheres comparable in number to those generated during continuous incubation with IGF-I and EGF. This effect could not be recapitulated by either a transient or continuous co-incubation of IGF-I with FGF-2. Therefore, the authors suggested that the continuous presence of IGF-I is not necessary for EGF-stimulated NSC proliferation (6). However, the results of that study were contradictory to those of Aberg et al. (7), who studied adult rat hippocampal NPs (7). Aberg et al. (7) showed that upon FGF-2 pretreatment in the presence of a low concentration of insulin [100 ng/ml], the expression of IGF-1R increases. They described distinct proliferative effects of FGF-2 (without IGF-I addition) and IGF-I (without FGF-2 addition) and showed that the effects were additive when both FGF-2 and IGF-I were used together (7).

The apparent contradiction between these studies, apart from the distinct cell types used, could be due to the presence of insulin in the culture medium, which will activate the IGF receptor system. As the majority of NP studies use a superphysiological concentration [25 μg/ml] of insulin in the culture medium that activates IGF-1R signal transduction, the role of IGF-1R signaling is generally unappreciated. Specifically, in postnatal NPs additional studies are needed to assess the separate effects of IGF-1R signaling from EGF receptor signaling. Moreover, if the IGFs increase the proliferation and survival of NPs, then it is important to evaluate the role of the IGFs in the context of developmental brain injuries where disturbances in IGF signaling may perturb normal brain development or recovery from injury. Thus, the focus of this study was to test the hypothesis that IGF-1R is necessary for the expansion of NPs following brain injury in the presence of EGF.

## Materials and Methods

### Neonatal hypoxia–ischemia

All animal work was approved by the institutional animal care and use committee guidelines of the New Jersey Medical School. Timed pregnant Wistar rats (Charles River Laboratories, Charles River, DE, USA) were housed on a 12 h light/dark cycle at 25°C with Purina rodent chow (catalog #5001). Following a normal delivery, the litter size was culled to 12 pups per litter. Neonatal H–I was induced on postnatal day 6 (P6) rat pups [P0 as day of birth] by a permanent right common carotid artery [CCA] cauterization followed by systemic hypoxia. Briefly, P6 rat pups were anesthetized with isoflurane [5% induction and 3% maintenance] following which a midline neck incision was made. The right CCA was separated from the vagus nerve and ligated using a bipolar cauterizer (Malis bipolar cauterize and bipolar cutter, Codman, Randolph, MA, USA) at 10 V. The skin incision was sutured using 4-0 silk, and the pups were returned to the dam for 2 h. Prior to hypoxia, the pups were pre-warmed in jars partially submerged in a 37 C water bath for 20 min after which rat pups were subjected to systemic hypoxia in jars with humidified 8% O_2_/92% N_2_ for 75 min. Sham-operated control animals had their right CCA separated from the vagus nerve but it was not ligated, and they were subjected to hypoxia. Following the hypoxia, the animals were left in the jars for 15 min at normoxia after which they were returned to their cages.

### Primary neurosphere assay

After 3 days of recovery from H–I, P9 pups were decapitated, their brains removed, and neurospheres generated essentially as previously described (8). For experiments on neurospheres from untouched rats, P4 rat pups were used. Under aseptic conditions, a cut was made 2 mm from the anterior pole of the brain. A second cut was made approximately 3 mm posterior to the first cut. The hippocampus, corpus callosum, and the meninges were removed under the microscope. Using forceps, 12 o’clock and 3 o’clock incisions were made, and the region enclosed between the cortex and the ventricle containing the SVZ was removed and placed in fresh PGM [1 mM MgCl_2_, 0.6% Dextrose in PBS, pH 7.3]. The tissue was mechanically dissociated using forceps and then enzymatically by the addition of 0.05% Trypsin/EDTA at 37°C for 7 min. The trypsin was inactivated by the addition of an equal volume of newborn calf serum, and the tissue was resuspended in ProN media [DMEM/F12 1:1 media containing 10 ng/ml d-biotin, 25 μg/ml insulin, 20 nM progesterone, 100 μM putrescine, 5 ng/ml selenium, 50 μg/ml apo-transferrin, 50 μg/ml gentamycin], and triturated in proN. The triturated suspension was passed through a 40 μM Nitex screen, and the cells were collected by centrifugation at 200 × *g* for 2 min and washed with ProN. The cells were counted with 0.1% Trypan blue dye under a hemocytometer and plated at 5 × 10^4^ cells/ml in ProN media containing 25 ng/ml insulin and supplemented with 2 ng/ml EGF, 1 ng/ml FGF-2, 15 ng/ml IGF-I, 28 ng/ml IGF-II, or combinations as stated. The cells were cultured at 37°C in 5% CO_2_ incubators and fed every 2 days by removing approximately half of the media and replenishing with fresh media.

### Secondary neurosphere propagation

Primary neurospheres were collected from 12-well plates after 6 days *in vitro* and pelleted by centrifugation at 12,000 rpm for 5 min. The neurospheres were enzymatically dissociated for 5 min at 37°C in a 0.01% trypsin/EDTA solution in GHCKS buffer (11 mM glucose, 20 mM HEPES, 10 mM citrate, 4 mM KCl, 110 mM NaCl, and 0.002 g/l phenol red). Trypsin was inhibited by adding 10% FBS in ProN media. For spheres that were generated for the early growth response factor-1 (EGR-1) experiment, Accutase was used to dissociate the primary spheres. The spheres were dissociated by trituration in ProN media using progressively less media and smaller pipette tips. The number of viable cells was determined in a hemocytometer via exclusion of 0.1% trypan blue dye. The cells were plated into a plastic 12-well tissue culture plate at a density of 5 × 10^4^ viable cells/ml in ProN media supplemented with growth factors as per experimental conditions. Cell cultures were fed every 2 days by removing approximately half the media and replacing with an equal volume of fresh media.

### Neurosphere quantification

A neurosphere was defined as a free-floating cluster of at least 25 μm in diameter. Prior to counting the spheres, the plates were shaken to ensure uniform distribution of the spheres in the well. The number of neurospheres in five random fields under 4× was determined for each well. The total number of neurosphere producing cells, the NPs in the population, was extrapolated from the average number of spheres per field, area of the field, and area of the well. The cells were plated at 5 × 10^4^ cells/ml and at 2 ml/well in a 6-well tissue culture dish for each condition. For neurosphere volumetric measurements, digital photographs were captured from at least 10 neurospheres from each condition (*n* = 6 SVZ per condition) and volumes calculated using IP Lab 3.6 software.

### Propidium iodide labeling for flow cytometry analysis of cell cycle

Neurospheres were dissociated by enzymatic and mechanical dissociation in 0.05% trypsin-EDTA, fixed in 70% ethanol, and then stored at −20°C until analysis. Cells were incubated with RNase 1 (Sigma, St. Louis, MO, USA) for 15 min and then stained with 50 mM propidium iodide (PI). PI is a fluorescent dye that intercalates into DNA, thus, the DNA content of a cell correlates with fluorescence intensity, providing a profile of cells in different phases of the cell cycle. PI stained cells were characterized using a Becton Dickinson FACS scan and the data acquired using CellQuest™ software (Becton Dickinson, Franklin Lakes, NJ, USA) and analyzed using ModFit™(Verity Software House, Topsham, ME, USA). ModFit uses a robust automatic analysis engine to detect peak and to identify ploidy patterns that correspond to different phases of the cell cycle.

### Western blot analyses

Total cell lysates from neurospheres were washed in ice-cold PBS and isolated in sodium dodecyl sulfate (SDS) buffer (62.5 mM Tris–HCl, 2% SDS, 10% glycol, 50 mM DTT, 1/100 protease inhibitor cocktail, 1 mM Na3VO4, and 1 mM NaF). Lysates were briefly sonicated and then subjected to a protein assay (Bio-Rad, Hercules, CA, USA). A total of 15–30 μg of cell lysates were boiled at 100°C for 5 min and resolved on 7, 10, or 4–12% mini gels by SDS polyacrylamide gel electrophoresis (SDS-PAGE). Separated proteins were electrotransferred to nitrocellulose membranes and blocked in 5% milk in TBS-1% Tween buffer for 1 h at room temperature. Membranes were incubated with primary antibodies overnight at 4°C (1:500, pHistone 3; 1:5000, β-actin; 1:250 for all other antibodies). The following day, membranes were washed 3 × 5 min in TBS-1% Tween and incubated with secondary antibodies, HRP-conjugated goat anti-mouse or goat anti-rabbit antibodies (1:5000) for 1 h at room temperature. The detection of HRP-conjugated secondary antibodies was performed with ECL (Perkin Elmer, Boston, MA, USA) using the Ultra-LUM imaging device (Claremont, CA, USA).

### Quantitative real-time PCR

RNA was isolated and reverse transcribed to cDNA using the iScript kit from Bio-Rad. Q-PCR was performed on the samples as described in Ref. (9) using beta actin as the internal control and with Quantitect real-time primers for EGR-1 (QT00385546).

## Results

### The increase in neurosphere number and size following neonatal H–I requires EGFR activation together with IGF-I co-signaling.

**Figure 1:**
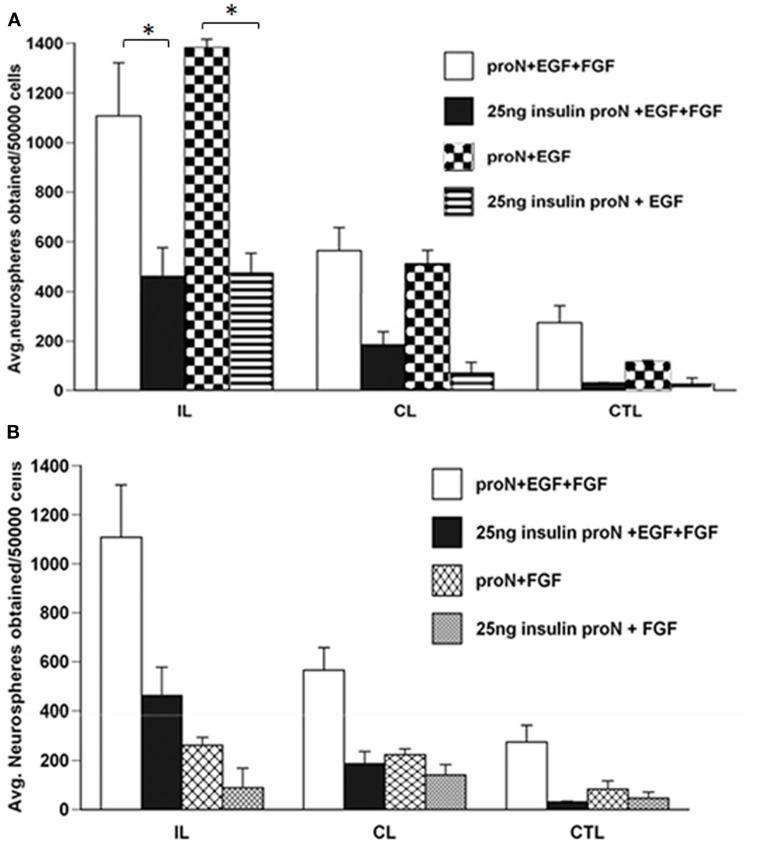
**The increase in neurosphere number after neonatal H–I requires EGFR activation together with IGF-R co-signaling**. SVZ cells were isolated from ipsilateral (IL), contralateral (CL), and sham-operated control (CTL) at 3-day recovery from H–I. The dissociated cells were cultured in media containing 25 μg/ml “high” insulin or 25 ng/ml “low” insulin media supplemented with 2 ng/ml EGF **(A)** or 1 ng/ml FGF-2 **(B)** for 7 days. The numbers of neurospheres obtained per 50,000 cells plated was counted. *p < 0.05 by MANOVA and Tukey’s *post hoc* tests. Data represent the mean number of neurospheres ± SEM from three experiments with six animals per experiment.

**Figure 2:**
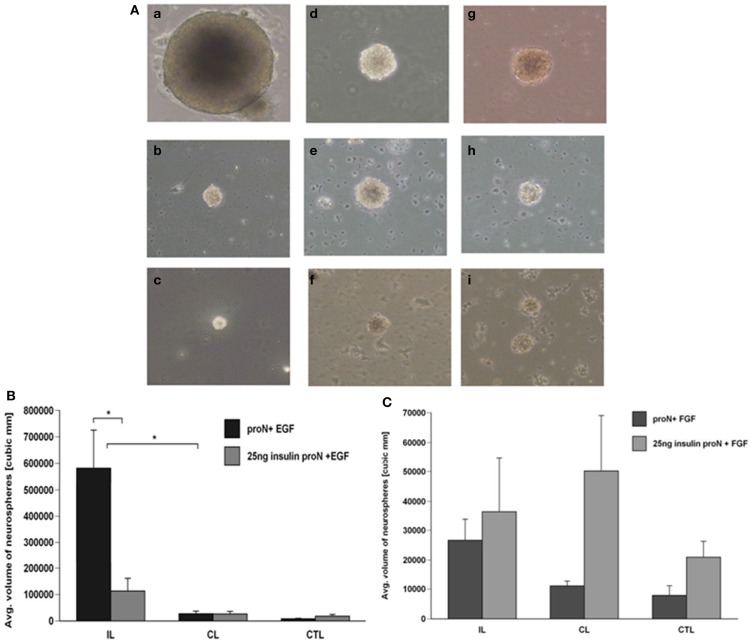
**Neurospheres grow larger in the presence of IGF-R signaling together with EGF but not FGF-2**. **(A)** Neurospheres were generated from ipsilateral (a–c), contralateral (d–f), and sham-operated control (g–i) SVZ at 3-day recovery from H–I and cultured in 25 μg/ml insulin[“high” insulin proN] supplemented with 2 ng/ml EGF (a, d, g); 25 ng/ml insulin[“low” insulin] supplemented with 2 ng/ml EGF (b, e, h) or 1 ng/ml FGF-2 (c, f, i) for 7 DIV. **(B)** The average number of neurospheres obtained per 50,000 SVZ cells was quantified for EGF in “high” and “low” insulin. **(C)** Quantification of the average size of neurospheres obtained with EGF in “high” and “low” insulin from ipsilateral (IL), contralateral (CL), and control hemispheres (CTL). *p < 0.05 by MANOVA and Tukey’s *post hoc* tests. Data represent mean number/size of neurospheres + SEM from three experiments with six animals per experiment.

Our previous studies have demonstrated that there is an increase in the number and size of neurospheres generated from the SVZ following H–I injury. This increase in neurosphere size and number occurs in response to EGF but cannot be recapitulated by FGF-2 (1). The serum free media for SVZ NPs has superphysiological levels of insulin that can activate the IGF-1R in addition to the insulin receptor (IR). Considering the results of the studies reviewed in the Introduction together with our data, we hypothesized that IGF-1R is necessary for the expansion of NPs following brain injury in the presence of EGF. Therefore, we performed experiments to evaluate the necessity of IGF-1R signaling by lowering the insulin concentration in the media, to levels that only stimulate IR, from 25 μg/ml “high” to 25 ng/ml “low” in NPs when exposed to EGF, FGF-2, or both. We found that this manipulation reduced the number of neurospheres that were obtained from the ipsilateral hemisphere cultured in low insulin media compared to high insulin media in the presence of EGF by 2.5-fold (Figure 1A). Similarly, reducing the concentration of insulin in the medium affected the size of neurospheres. Neurospheres produced from the ipsilateral hemisphere in EGF containing “low” insulin media were 3-fold smaller than “high” insulin media [*p* < 0.05] (Figures 2A,B).

As earlier studies had shown that FGF-2 promoted NP proliferation, we evaluated the necessity of IGF-1R signaling in the FGF-2-mediated response of NPs following injury. Again, lowering the insulin concentration from 25 μg/ml to 25 ng/ml, reduced the number of neurospheres obtained from the ipsilateral hemisphere by 3-fold in the presence of FGF-2. However, it is important to note that the numbers of neurospheres generated in the presence of FGF-2 were significantly lower compared to EGF even in the presence of “high” insulin levels (Figure 1B). The size of neurospheres obtained in FGF-2-media was not different when cultured in the presence of 25 μg/ml “high” insulin or 25 ng/ml “low” insulin media (Figures 2A,C).

We had previously shown that neurospheres grow larger in size and number from the ipsilateral hemisphere compared to contralateral and sham-operated control SVZ in EGF supplemented media (8). Interestingly, the neurospheres obtained when grown in EGF were significantly larger even in the presence of 25 ng/ml “low” levels of insulin (Figures 1A and 2A,B). By contrast, there were no significant differences in neurosphere number and size in FGF-2 supplemented media containing 25 ng/ml “low” insulin (Figures 1B and 2A,C). These data cannot be attributed to differences in survival as NPs maintained in low insulin and EGF for 7 days retained the capacity to form neurospheres when transferred to high insulin containing medium and EGF (data not shown), and similar results are obtained using this same experimental design with mouse NPs (9).

### IGF-II is a better mitogen than IGF-I for growth and self-renewal of NPs

**Figure 3:**
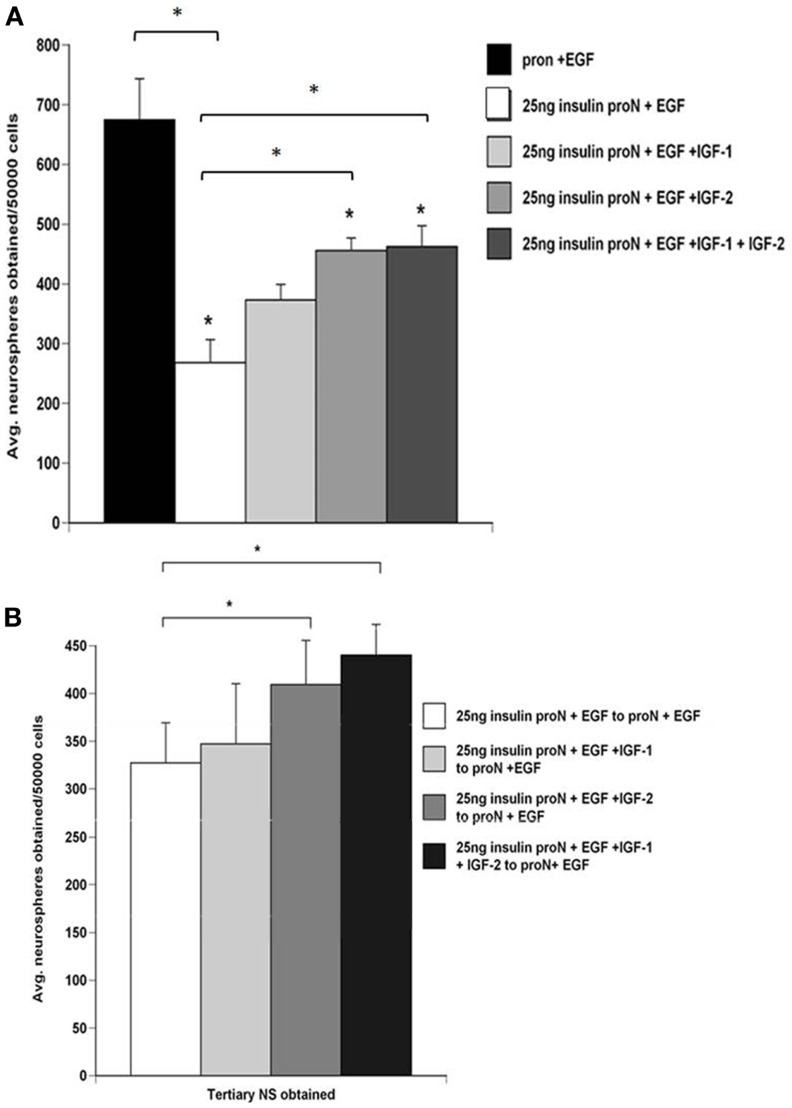
**NPs cultured in IGF-II generate more neurospheres which promotes their self-renewal better than spheres grown in IGF-I**. SVZ cells from control rat brains were cultured in 25 μg/ml “high” insulin or 25 ng/ml “low” insulin media containing 2 ng/ml EGF supplemented with IGF-I (15 ng/ml), IGF-II (28 ng/ml), or both. **(A)** Quantification of the average number of neurospheres obtained per 50,000 SVZ cells. **(B)** Numbers of tertiary neurospheres obtained upon passaging to 25 ng/ml insulin media containing 2 ng/ml EGF. *p < 0.05 by MANOVA and Tukey’s *post hoc* tests.

Emerging data indicate that NPs express both the IGF-I receptor as well as the insulin receptor A isoform, through which IGF-II, but not IGF-I, signals. Therefore, to determine whether IGF-I and IGF-II affect SVZ NP proliferation and self-renewal differently, we dissociated primary rat neurospheres and cultured them to generate secondary neurospheres in 25 ng/ml “low” insulin containing media supplemented with EGF and further supplemented with IGF-I alone, IGF-II alone, or the combination of IGF-I and IGF-II. IGF-I and IGF-II were used at their Kds for the IGF-1R. Supplementing the medium with IGF-I or IGF-II significantly increased the number of neurospheres generated vs. “low” insulin media with EGF. The combination of both IGF-I and IGF-II produced even greater growth than IGF-I alone (Figure 3A). Interestingly, IGF-II generated more (*p* < 0.05) neurospheres than IGF-I supplemented media. There was no significant increase between IGF-II and a combination of IGF-I and IGF-II on neurosphere numbers obtained (Figure 3A).

To establish whether these growth factors affected SVZ NP self-renewal independently or in combination, secondary neurospheres generated in 25 ng/ml “low” insulin containing media supplemented with EGF only or with IGF-I, IGF-II, or the combination were passaged and then maintained in 25 μg/ml “high” insulin media with EGF. Interestingly, spheres grown in IGF-II generated more tertiary spheres than spheres grown in IGF-I (Figure 3B). The number of tertiary spheres generated from cells exposed to IGF-I and EGF with “low” insulin was not significantly different from those exposed to “low” insulin media containing EGF alone in the absence of exogenous IGF-I (Figure 3B). In previous studies, we evaluated how the IGF ligands affected sphere potentiality. Spheres that were propagated in EGF containing medium supplemented with either IGF-I, IGF-II, or IGF-I + IGF-II were differentiated and stained for markers of neurons or glia. Spheres propagated in IGF-I and EGF were rarely tripotential. By contrast, the percentage of tripotential neurospheres was greatest in IGF-II supplemented medium (9).

### IGF-1R and EGFR co-stimulation is necessary for cell cycle progression

**Figure 4:**
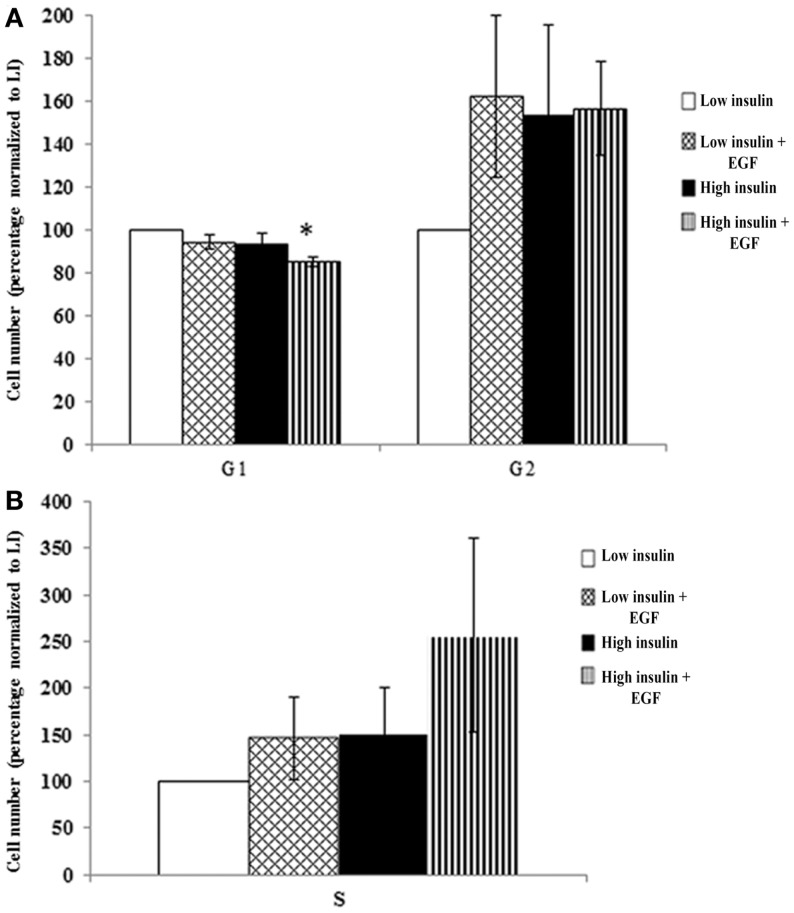
**IGF-1R activation together with EGFR co-signaling decreases G1 that leads to a trend in cell cycle progression**. Neurospheres were cultured in “high” insulin media for 3 DIV, growth factor deprived for 18 h and then stimulated with either “low” or “high” insulin media with or without EGF for 72 h prior to cell cycle analysis using propidium iodide staining and flow cytometry. G1 and G2 **(A)** and S phase **(B)** were normalized to “low” insulin media (*n* = 3) averaged ± SEM, *indicates significance via ANOVA with Tukey’s *post hoc*.

To determine whether cooperatively stimulating the IGF and EGF receptors was necessary for cell cycle progression, and thus the expansion of the population in culture, we assessed cell cycle using flow cytometry. Secondary neurospheres were propagated for 3 DIV in “high” insulin media with EGF. The neurospheres were then starved for 18 h and stimulated for 72 h with either “low” insulin or “high” insulin, with or without EGF. An analysis of cells in S, G1, and G2 phases of the cell cycle using PI showed that there was a significant decrease in percentage of cells in G1 only when both IGF-1R and EGFR were stimulated (Figure 4A). There was a trend toward an increase in the proportion of cells in both S and G2 phases in the same, “high” insulin with EGF condition (Figures 4A,B). The variability in the number of cells found in S and G2 phases likely reflects the rapidity with which cell traverse these phases in contrast to the time a cell spends in G1, even when normally cycling. Using NP cultures, it is impossible to synchronize the cells to better study these shorter cell cycle phases as for traditional 2D culture conditions.

**Figure 5:**
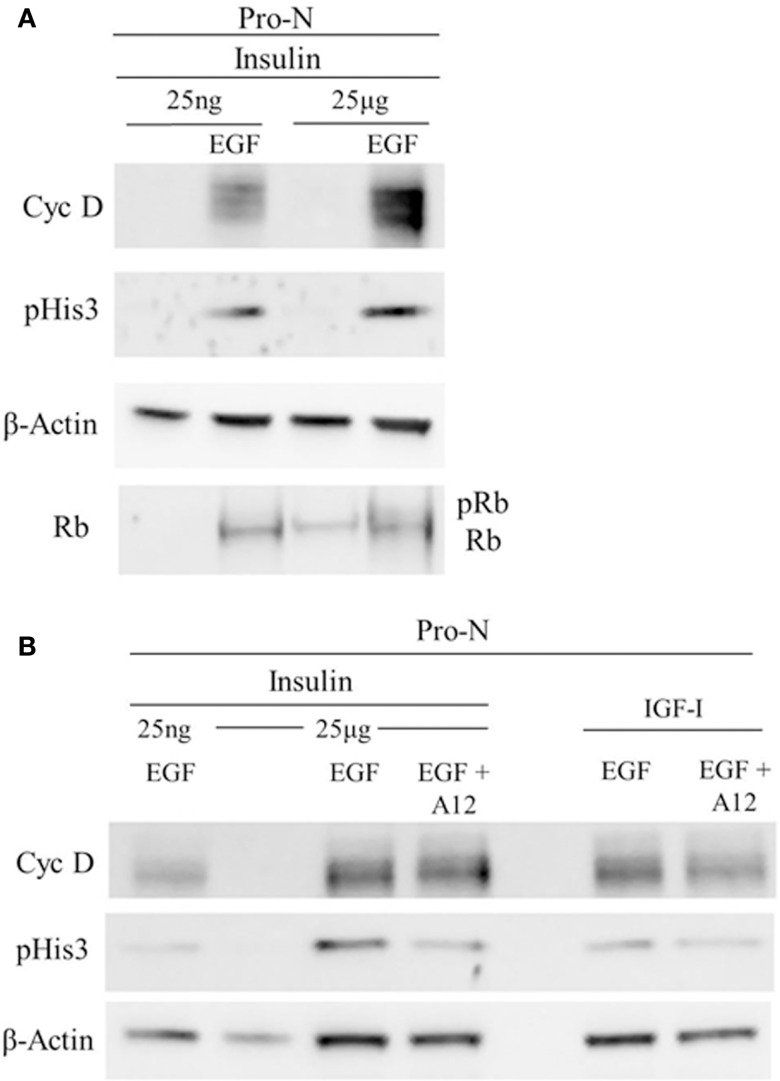
**Cell cycle progression marker expression is dependent on IGF-1R activation together with EGFR co-stimulation**. Neurospheres were cultured for 3 DIV in “high” insulin, starved for 18 h, and then stimulated with “high” [25 μg/ml] insulin or “low” [25 ng/ml] insulin, with or without EGF **(A)** or with IGF-I with or without EGF **(B)** for 72 h. IGF-1R blocking antibody, A12, was used to block both IGF-I and “high” insulin stimulation of IGF-1R**(B)**.

Cell cycle progression was further analyzed using Western blot for several key cell cycle proteins (Figure 5). These analyses revealed a large increase in cyclin D1 when both IGF-1R and EGFR were stimulated compared to “high” insulin alone (IGF-1R stimulation) or “low” insulin with EGF (EGFR stimulation). These conditions also increased levels of phosphorylated Rb. Taken together, these results support the conclusion that IGF-1R and EGFR cooperated to promote G1 progression through the G1/S transition. An increase in phosphorylated histone 3 suggests further progression into the G2 phase of the cell cycle. To further test whether the increase in phosphorylated histone 3, and cyclin D1 were dependent on the activation of the IGF-1R, we incubated the cells in an IGF-1R blocking antibody, A12, to block the action of “high” insulin or IGF-I on the IGF-1R (Figure 5). When A12 was present, both cyclin D1 and phosphorylated histone 3 levels decreased.

### EGF and IGF-I together increase levels of early growth response factor-1 (EGR-1)

**Figure 6:**
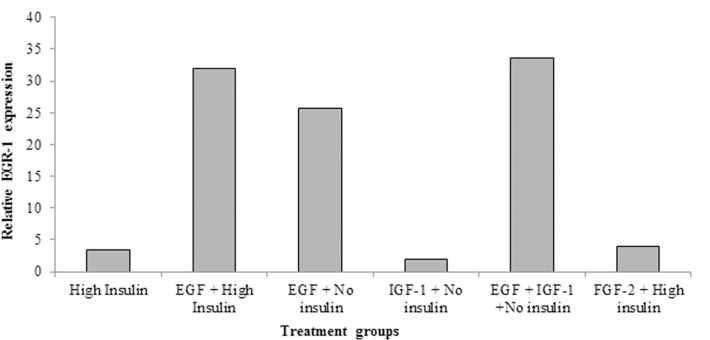
**EGF strongly induces EGR-1 expression in neural precursors**. Primary neurospheres were generated from P4–5 pups in standard medium. After passaging, the cells were shifted to medium containing “High” insulin (25 μg/ml), EGF (2 ng/ml) + “high” insulin, EGF (2 ng/ml) + no insulin, IGF-I (15 ng/ml) + no insulin, or EGF (2 ng/ml) + IGF-I (15 ng/ml). Cells were grown for 6–7 days, whereupon total RNA was isolated and relative levels of EGR-1 mRNA measured by QRT-PCR. Values are the averages of two independent experiments.

EGR-1 regulates the expression of many genes including the EGF receptor. Therefore, we hypothesized that IGF-I might induce the expression of EGR-1 to promote EGF signaling. Neurospheres were grown in “high” insulin medium with EGF for 6–7 days and then passaged. The cells were then incubated in growth factor free medium for 16 h and then stimulated with EGF alone (no insulin), IGF-I alone (no insulin), both EGF and IGF-I (no insulin), EGF + “high” insulin or “high” insulin. The relative expression of EGR-1 was highest when both EGF and IGF-I were added to the media, however, contrary to our prediction, IGF-I alone only weakly induced EGR-1 expression (Figure 6) (Values averaged from two independent experiments).

## Discussion

It has been well-established that NPs can be cultured *in vitro* in the presence of EGF and/or FGF-2. Moreover, infusing these growth factors into the ventricles increases the proliferation of periventricular SVZ NPs (10, 11). Whereas, these two growth factors are widely viewed as necessary for neural development (10), the importance of the IGFs has been under appreciated, despite the fact that the IGFs are abundant in the immature brain where IGF-I is produced by neurons and IGF-II by the choroid plexus and meninges (12–14). Likely the IGFs have been ignored because the culture medium used to propagate NPs has high levels of insulin, which will stimulate IGF system receptors, thus, masking the essential roles of the IGFs and their receptors in NP cell biology.

Our data show clearly that the expansion of SVZ NPs following brain injury requires EGFR and IGF-1R co-signaling. Previously, we established that EGFR signaling is necessary for SVZ NP expansion following brain injury by pharmacologically inhibiting EGFR activation and effectively down-regulating the NP expansion *In vitro*. Furthermore, we showed that overexpressing a constitutively active EGFR within NPs was sufficient to increase entry of these cells into the S phase of cell cycle (1). But those studies were all conducted under conditions where there was concurrent IGF-1R stimulation. Here, we sought to determine the role of IGF-1R signaling in the EGF-mediated response of SVZ NPs. To do so, we replaced the supraphysiological levels of insulin [25 μg/ml] in our base culture media with a physiological concentration of insulin [25 ng/ml] that would activate the insulin receptor but not the IGF-1R, and we evaluated the effect of this manipulation on NP growth, survival, and self-renewal. We report that: (1) The increase in neurosphere number and size that occurs in response to neonatal H–I requires IGF-1R activation together with EGFR co-signaling; (2) NPs cultured in IGF-II generate more neurospheres and self-renew better than NPs grown in IGF-I; and (3) IGF-1R activation together with EGFR co-signaling stimulates cell cycle progression.

Reminiscent of our data, an earlier study by Lin et al. (15), showed that a single intraventricular injection of IGF-I immediately after neonatal H–I reduced the extent of damage and increased the number of proliferating cells within the SVZ (15). The fact that IGF-I was neuroprotective supports the view that beneficial effects of the IGFs in the context of neonatal H–I will extend beyond their effects on the NPs of the SVZ. For example, Wood et al. (16) demonstrated that infusing IGF-I into neonatal rat pups acutely after H–I increased the number of oligodendrocyte progenitor cells (OPC) in white matter (16).

Other studies conducted on primary OPCs are informative in helping to understand how two mitogens can promote cell cycle progression in NPs. These studies have revealed how two essential growth factors for the early OPCs, FGF-2 and IGF-I, can work synergistically to promote progression through cell cycle. In OPCs, FGF-2 activates p42/p44 to reduce the levels of the Cdk inhibitor p27 (Kip1), while IGF-I promotes the phosphorylation of GSK-3β via PI3-kinase to jointly increase cyclin D1 accumulation within the nucleus and to promote S phase transition. In our cell cycle analyses, IGF-1R stimulation with insulin decreased the percentage of cells in G1 with a trend toward an increase in S and G2. This correlated with an increased phosphorylation of Rb and increased levels of cyclin D1, indicators of G1 progression and essential for pushing cells past the G1/S transition. When IGF-1R activation was blocked using the A12 antibody, these alterations in cell cycle protein expression were inhibited indicating a need for receptor co-stimulation for cell cycle progression. Thus, it is likely that similar signal transduction mechanisms are in play in the NPs as in the OPCs. Similar studies on mouse NPs that compared NP growth when maintained in “high” insulin vs. IGF-II showed more progenitors in the “high” insulin group, indicating that the “high” insulin stimulates more progenitors to divide (17). Additional data are needed; however, mechanistic studies are difficult to complete using neurospheres because of the heterogeneity of the cell population in a neurosphere. Recent flow cytometry studies indicate that there are likely to be as many as 12 different NPs within a medium-sized neurosphere (18). This heterogeneity likely contributed to the failure to achieve a significant effect in the proportion of cells in S phase between groups.

Cross-talk between IGF-1R and EGFR has been reported in breast, lung, and prostate cancer cells (19, 20) as well as in COS cells (21), which has stimulated interest in understanding the cooperativity between these receptors. Roudabush et al. (21) showed that activating the IGF-I receptor lead to a matrix metalloprotease-dependent release of HB-EGF that caused EGFR transactivation. The transactivated EGFR accounted for IGF-I-stimulated recruitment of adaptor molecules such as Shc, Grb2, SOS, and the subsequent activation of the Ras/Raf/MEK/ERK signaling pathway (21). Similar signaling was implicated in the studies by Jones et al. (19). They reported that the initial growth arrest produced by an EGFR inhibitor gefinitib (ZD 1839; IRESSA) was lost within months of therapy in breast and prostate cell lines. The acquired resistance to the drug was due to the formation of hybrid EGFR/IGF-1R receptors (19). Riedemann et al. (20) showed direct hybrid dimerization between the two receptors using reciprocal co-immunoprecipitation (20).

This study, together with our earlier studies, demonstrates that IGF signaling is important for the expansion of NPs observed after injury in response to EGF. NPs express both the IGF-1R and IR-A and our studies here on rat NPs and earlier studies on mouse NPs show that antagonizing the IGF-1R inhibits the growth of NPs (9). Additionally, IGF-1R stimulation with insulin results in neurospheres that contain more progenitors compared to ones cultured with IGF-II (17). We demonstrate that more neurospheres are generated in EGF containing media supplemented with IGF-II compared to IGF-I. While a combination of IGF-I and IGF-II is able to recapitulate the growth observed in “high” insulin media, our collective results from this paper and previous studies indicate that the spheres produced under these two sets of growth conditions are not comprised of the same cell populations as IGF-II exerts effects beyond those of IGF-1. Co-stimulation of EGFR and IGF-1R result in the “typical” growth that is observed in neurosphere cultures.

In a recent paper, Scafidi et al. (22) showed that stimulating the EGFR through intranasal heparin-binding EGF administration was both neuroprotection and pro-regenerative in that it enhanced the generation of new oligodendrocytes from progenitors and promoted functional recovery (22). They concluded that stimulating the EGFR in oligodendrocyte progenitors at a specific time after injury was a clinically viable treatment for babies born prematurely and who sustain white matter injury. However, their studies were conducted under conditions where the IGF-1R was activated. Though their results were impressive, it is possible that an even better outcome could have been achieved by also stimulating the IGF-1R. Thus, understanding the coordinated signaling between IGFs and EGFs may be essential to establish a robust regenerative response from SVZ NPs following brain injury.

## Author Contributions

Dhivyaa Alagappan: Conception and design, collection of data, data analysis and interpretation, manuscript writing, and final approval of manuscript. Amber N. Ziegler: Conception and design, financial support, collection of data, data analysis and interpretation, manuscript writing, and final approval of manuscript. Shravanthi Chidambaram: Conception and design, collection of data, data analysis and interpretation, manuscript writing, and final approval of manuscript. Jungsoo Min: Conception and design, collection of data, data analysis and interpretation, manuscript writing, and final approval of manuscript. Teresa L. Wood: Administrative support, financial support, conception and design, manuscript writing, and final approval of manuscript. Steven W. Levison: Administrative support, financial support, conception and design, manuscript writing, and final approval of manuscript.

## Conflict of Interest Statement

The authors declare that the research was conducted in the absence of any commercial or financial relationships that could be construed as a potential conflict of interest.
